# The Question of Contagion in Cancer

**Published:** 1889-11

**Authors:** 


THE QUESTION OF CONTAGION IN CANCER.—The editor of N. Y. Med. Record says: Notwithstanding the unsatisfactory nature of the evidence which has been brought foward in support of the contagiousness of cancer there are not a few who hold to this view. Several have claimed to have discovered a pathogenic micro-organism of the disease but none of these discoverers has had the satisfaction of finding his results confirmed by other investigators. Inoculation experiments upon animals with cancer-juice and with cultures of the micro-organisms have also failed to give results which can in any sense be regarded as conclusive. Inoculation upon the human subject has been tried in two instances at least, and has apparently succeeded. The first case was that of Alibert who derided the theory of coniagion, and to show his belief in its falsity he inoculated himself with cancer-juice, and subsequently died of carcinoma. The second case was one reported by Hahn, who inoculated the healthy breast with cancerous material taken from a tumor of the other breast. At the womon's death a cancerous growth was found at the site of inoculation.

